# Rhabdovirus Infection Is Dependent on Serine/Threonine Kinase AP2-Associated Kinase 1

**DOI:** 10.3390/life10090170

**Published:** 2020-08-30

**Authors:** Jun Luo, Yue Zhang, Yang Wang, Qing Liu, Luman Chen, Boyue Zhang, Yongwen Luo, Shile Huang, Xiaofeng Guo

**Affiliations:** 1 College of Veterinary Medicine, South China Agricultural University, Guangzhou 510642, China; lj378483477@163.com (J.L.); yuezh1009@163.com (Y.Z.); jiusanwang93@163.com (Y.W.); LQ4468441@163.com (Q.L.); luman@stu.edu.cn (L.C.); zhangboyue163@163.com (B.Z.); ywluo@scau.edu.cn (Y.L.); 2 Department of Biochemistry and Molecular Biology, Louisiana State University Health Sciences Center, 1501 Kings Highway, Shreveport, LA 71130-3932, USA; shuan1@lsuhsc.edu; 3 Feist-Weiller Cancer Center, Louisiana State University Health Sciences Center, Shreveport, LA 71130-3932, USA

**Keywords:** rabies virus, AP-2-associated protein kinase 1, vesicular stomatitis virus, rhabdovirus, therapy

## Abstract

Rabies virus (RABV) causes a fatal neurological disease in both humans and animals. Understanding the mechanism of RABV infection is vital for prevention and therapy of virulent rabies infection. Our previous proteomics analysis based on isobaric tags for relative and absolute quantitation to identify factors revealed that RABV infection enhanced AP-2-associated protein kinase 1 (AAK1) in N2a cells. In this study, to further confirm the role of AAK1, we showed that RABV infection increased the transcription and expression of AAK1 in N2a cells. AAK1 knockdown significantly decreased RABV infection in both N2a and BHK-21 cells. AAK1 knockout inhibited RABV infection in N2a cells. Furthermore, inhibition of AAK1 kinase activity using sunitinib decreased RABV infection. However, AAK1 overexpression did not change RABV infection in vitro. Therapeutic administration of sunitinib did not significantly improve the survival rate of mice following lethal RABV challenge. In addition, AAK1 knockdown decreased infection in N2a cells by vesicular stomatitis virus, which is another rhabdovirus. These results indicate that rhabdovirus infection is dependent on AAK1 and inhibition of AAK1 is a potential strategy for the prevention and therapy of rabies.

## 1. Introduction

Rabies, which is caused by rabies virus (RABV), is an ancient zoonosis of the central nervous system (CNS) that causes more than 55,000 human deaths each year [1]. Currently, there is no treatment for RABV infection once symptoms of the disease have become evident. However, rabies can be averted by effective and timely post exposure treatment, consisting of rabies vaccination and serotherapy [2]. Even so, rabies immune globulin used for PEP is expensive, so many people in developing countries cannot afford it. In addition, PEP has occasionally failed to prevent rabies-related deaths because of nonstandard and delayed treatment. Therefore, alternative approaches to the treatment of rabies are urgently required. RABV is a negative-stranded RNA virus in the genus *Lyssavirus* of the family *Rhabdoviridae*. The RABV genome is approximately 12 kb in size, and comprises five genes, which encode nucleoprotein (N), phosphoprotein, matrix protein (M), glycoprotein (G), and the RNA-dependent RNA polymerase (L) [3]. RABV first interacts with cellular receptors, including acetylcholine receptors, neural cell adhesion molecules, low-affinity neurotrophic receptors, and metabotropic glutamate receptor subtype 2 [4,5,6,7]. After RABV attaches to the cell membrane, a process of viral internalization is activated via the endocytic pathway and is mediated by clathrin and actin [8,9,10,11]. RABV moves to CNS through retrograde movement along the axon in acidic compartments [12]. In infected cells, ribonucleoprotein complexes (RNP) containing genomic RNA, N, L, and P are released and template genomic RNA replication and mRNA synthesis [13], which is regulated by M and G [14]. After replication, progeny RABV was assembled with genomic RNA and synthesized structural proteins in the Negri bodies and then, released via budding [15]. Moreover, pathogenic RABV infection can promote the survival of the infected neurons through inhibition of induction of apoptosis [16]. Any mediators that regulate the life cycle of RABV infection can be identified as novel targets for the treatment of rabies.

Previous studies have confirmed that clathrin-mediated endocytosis is regulated by adaptor protein 2 (AP2) [17,18] and Numb [19]. AAK1 phosphorylates the AP2 mu subunit [20] and subsequently mediates clathrin-mediated endocytosis [21,22]. In addition, AAK1 regulates Numb activity by phosphorylation and promoting coated pit maturation in clathrin-mediated endocytosis [23]. Thus, AAK1 also plays an important role in clathrin-mediated endocytosis. Studies have indicated that clathrin-mediated endocytosis in hepatitis C virus (HCV) infection is mediated by AAK1 [24,25]. Furthermore, treatment with pharmacological inhibitors of AAK1 inhibits the entry of HCV into host cells, thus, implicating AAK1 as a therapeutic target in HCV infection. A recent study based on high-throughput RNA interference assays confirmed that AAK1 plays a critical role in regulating the clathrin-mediated endocytosis of RABV [26]. Therefore, AAK1 is involved in the entry of viruses via its function of regulating clathrin-mediated endocytosis. In our previous study, we showed that RABV infection increases AP-2-associated protein kinase 1 (AAK1) expression in mouse neuroblastoma (N2a) cells through proteomics analysis based on isobaric tags for relative and absolute quantitation (iTRAQ) [27]. In this study, we further confirmed the role of AAK1 in RABV infection. We also showed that AAK1 plays a critical role in infection by vesicular stomatitis virus (VSV), which is another rhabdovirus. These results confirm that AAK1 is a potential drug target for the treatment of RABV infection.

## 2. Material and Methods

### 2.1. Cells, Plasmids, Viruses, Antibodies, and Reagents

N2a cells were purchased from Wuhan Institute of Biological Products (Wuhan, China) and cultured in RPMI 1640 (Gibco, Suzhou, China) supplemented with 10% fetal bovine serum (FBS) (Gibco, Grand Island, New York, NY, USA). Baby hamster kidney cells (BHK-21) (Wuhan Institute of Biological Products, Wuhan, China) were maintained in Dulbecco’s modified Eagle’s medium (DMEM) (Gibco, Suzhou, China) supplemented with 10% FBS. The pSpCas9 (BB)-2A-Puro (PX459) V2.0 construct was a gift from Feng Zhang (Addgene plasmid # 62988; http://n2t.net/addgene:62988; RRID:Addgene_62988) [28]. The pcDNA3.1 (+) plasmid was purchased from Invitrogen (Carlsbad, CA, USA). The RABV rHEP-GFP strain, which carries green fluorescent protein (GFP) between N and P genes, was constructed as reported previously [29]. The RABV CVS-11 strain (a gift from Dr. Xianzhu Xia, Academy of Military Medical Sciences, Beijing, China) was propagated in N2a cells. VSV (a gift from Fuyan Chen, Nanjing Agricultural University, Nanjing, China) was propagated in BHK-21 cells. Fluorescein isothiocyanate (FITC)-conjugated anti-RABV-N antibodies were purchased from Fujirabio Inc. (Malvern, PA, USA). Anti-RABV-M antibody was prepared in our laboratory (unpublished data). Anti-AAK1 antibody was purchased from Abcam (Shanghai, China). Cy3-conjugated anti-rabbit IgG was purchased from Beyotime Biotechnology (Shanghai, China). Sunitinib (Dingguo, Beijing, China) was dissolved in dimethyl sulfoxide (DMSO) and then, diluted in PBS for in vivo use.

### 2.2. Animals

Female Kunming (KM) mice (Center for Laboratory Animal Science of the Southern Medical University, Guangzhou, China) were housed at the Laboratory Animal Center of the South China Agricultural University. All animal experiments were approved by the Ethics Committee for Animal Experiments of the South China Agricultural University (ethics committee approval number: 2019e001).

### 2.3. RABV Titration

Rabies virus titrations were conducted using direct fluorescent antibody assays (dFA) as previously described [30]. Briefly, N2a cells cultured in 96-well plates were inoculated with 10-fold serial dilutions of CVS-11 and rHEP-GFP strains in RPMI 1640 medium. The cells were cultured at 37 °C for 2 days. The supernatants were then discarded and cells were fixed with 80% acetone for 30 min at −20 °C. Cells were washed three times with PBS and stained with FITC-labeled anti-RABV-N antibodies for 60 min at 37 °C. Antigen-positive foci were observed under a fluorescence microscope (AMG, Washington, DC, USA) and virus titers were calculated as focus-forming units (FFU) per milliliter (FFU/mL) using the Karber method.

### 2.4. Quantitative Real-Time PCR

Infected N2a cells were harvested at 24 h post infection (hpi) and total RNA was prepared using TRizol reagent (Magen, Guangzhou, China) following the manufacturer’s instructions. Reverse transcription was performed using the Transcriptor First Strand cDNA Synthesis Kit (Vazyme Biotech, Nanjing, China). Each reaction was performed using SYBR Green Master Mix (Vazyme Biotech). Quantitative real-time PCR (qRT-PCR) was performed using a CFX connect Real-Time System (Bio-Rad, Hercules, CA, USA). The levels of AAK1 mRNA, VSV N mRNA, and RABV genomic RNA (gRNA) were normalized to glyceraldehyde-3-phosphate dehydrogenase (GAPDH). The sequences of the primers for amplification of AAK1 were as follows: forward 5′-AGTTTGCCCCCATAGCACTC-3′ and reverse primer 5′-CCTAGAGTGCCCACCTTGTG-3′. The sequences of the primers for amplification of VSV N mRNA were as follows: forward 5′-ACATATGGGAGAATGATCC-3′ and reverse primer 5′-TCTTGAGACTATGGTTCCG-3′. The sequences of the primers used for amplification of gRNA and GAPDH were as described previously [31].

### 2.5. Indirect Fluorescent Antibody Assay (iFA)

N2a cells in 96-well plates were infected with RABV for 24 h and then, cells were fixed in 80% acetone at −20 °C for 30 min and washed three times with PBS. Fixed cells were stained with anti-AAK1 antibodies at 37 °C for 1 h and washed three times with PBS. The cells were then stained with Cy3-conjugated anti-rabbit IgG at 37 °C for 2 h. After the incubation of anti-AAK1 antibodies, cells were stained with FITC-labeled anti-RABV-N antibodies for 1 h at 37 °C and with DAPI dihydrochloride (DAPI) at room temperature for 3 min in the dark. Cells were washed three times with PBS and antigen-positive foci (red and green) were observed under a fluorescence microscope (AMG).

### 2.6. Western Blot Analysis

Western blot was performed as previously described [32]. Briefly, cell lysates were separated by 12% sodium dodecyl sulfate-polyacrylamide gel electrophoresis and transferred to polyvinylidene difluoride membranes (Millipore, Bedford, MA, USA). The membranes were then probed with antibodies against AAK1, RABV-M or β-actin. Protein bands were imaged using a Fine-do X6 Chemiluminescent Imaging System (Tanon, Shanghai, China).

### 2.7. RNA Interference

N2a cells and BHK-21 cells were transfected with AAK1-targeting siRNAs using Lipofectamine 3000 Reagent (Invitrogen), according to the manufacturer’s protocol. The sequences of the AAK1-targeting siRNAs were as follows: AAK1-1 (UCAUAAAGCUGCAGAAGAUTT) and (CUGAAGAGCUGCUAAACAATT). A non-targeting (NC) siRNA (UUCUCCGAACGUGUCACGUTT) was used as a negative control. The siRNAs were synthesized by Sangon Biotech (Shanghai, China). Cells were infected with the virus and checked for the presence of RABV at 24 hpi under a fluorescence microscope. Supernatants were harvested to determine the virus titers, as described above. At 24 hpi, cells were collected to investigate the expression of AAK1 and RABV-M by Western blot analysis and gRNA levels were analyzed by qRT-PCR.

### 2.8. Generation of AAK1-Deficient N2a Cells Using CRISPR-Cas9

Three AAK1-targeting guide RNAs were designed based on NCBI reference sequences (Gene ID: 269774): AAK1-RNA1, 5′-CACCGTGCTACTTCACTTTGCCGTT-3′, AAK1-RNA2, 5′-CACCGTGTGAAAAAGACCCAGCCAA-3′, AAK1-RNA3, 5′-CACCGCTTCAATCGCACCCCGCCAG-3′, and cloned into plasmid pSpCas9 (BB)-2A-Puro (PX459) V2.0 as described previously [28]. N2a cells were transfected with the constructed recombinant plasmids or empty vector using Lipofectamine 3000 transfection reagent according to the manufacturer’s instructions. At 36 h after transfection, supernatants were removed and replaced with fresh RPMI 1640 medium supplemented with 10% FBS containing 3 μg/mL puromycin (Cayman Chemical, Ann Arbor, MI, USA). The cells were cultured for a further 48 h and before the surviving N2a cells were trypsinized, and detached cells were seeded in 96-well plates in high dilution for further culture. Cells that proliferated from a single cell were amplified and AAK1 expression was analyzed by Western blotting with the anti-AAK1 antibody.

### 2.9. Pharmacological Treatment of N2a Cells

N2a cells were seeded in 6-well plates and were treated with 10 μM sunitinib (pharmacological inhibitor of AAK1) or 2 μL DMSO at 37 °C for 2 h before infection with the RABV CVS-11 strain at a multiplicity of infection (MOI) of 1. The supernatants were harvested at 24 hpi and the virus titers were determined using dFA with FITC-labeled anti-RABV-N antibodies as described above.

### 2.10. AAK1 Overexpression in N2a Cells

The *AAK1* gene was amplified from N2a cells by RT-PCR using the primers AAK1-F: 5′-CCCCGAATTCATGAAGAAGTTTTTCGACTCC-3′ and AAK1-R: 5′-TTTTCTCGAGCTACAGGTCTATGAGCTGATC-3′ designed based on the NCBI reference sequence (GenBank No.: NM_001040106.2). The *AAK1* gene was cloned into pcDNA3.1 (+) using the *EcoR*I and *Xho*I restriction enzyme sites and the product was designated p3.1-AAK1. N2a cells were seeded in 6-well plates and transfected with p3.1-AAK1 or pcDNA3.1 (+) using Lipofectamine 3000 transfection reagent according to the manufacturer’s instructions. At 24 h post transfection, cells were infected with the RABV CVS-11 strain at an MOI of 1 and cultured for a further 24 h at 37 °C. Supernatants were harvested to determine RABV titers as described above.

### 2.11. VSV Titration

VSV titers were determined as described previously [33,34]. Briefly, BHK-21 cells were seeded in 96-well plates and inoculated with 10-fold serial dilutions of VSV in DMEM. Cells were then cultured at 37 °C for 2 days before the culture medium was discarded and cells were fixed with 80% acetone for 30 min at −20 °C. Cytopathic effects were observed after 36 h inoculation and scored under a standard light microscope. The virus titers were calculated and expressed in cytopathy-forming units (CFU) per milliliter (CFU/mL) using the Karber method [35].

### 2.12. Challenge Protection Assay

Before the challenge assay, the toxicity of sunitinib was investigated in vivo. KM mice were treated with 0.10 mg sunitinib by intramuscular (i.m.) injection. PBS treatment was used as a control. Each group contained 6 mice. The body weights of mice were monitored every day for 15 days after treatment. For the challenge assay, KM mice were intramuscularly (i.m.) inoculated at the gastrocnemius muscle of the right leg with 3.16 × 10^5^ FFU of CVS-11. At 2 days post infection, mice were treated with 0.10 mg sunitinib in PBS via the i.m. route at the gastrocnemius muscle of the right leg. Survival rates were recorded daily for 3 weeks and data were analyzed by the logrank Mantel–Cox test using GraphPad Prism software.

### 2.13. Statistical Analysis

Data were presented as mean values ± standard deviation (SD) from three to four independent experiments, and analyzed using Prism 6 (GraphPad software, San Diego, CA, USA). Statistical significance was determined using Student’s *t*-test, one-way ANOVA with the Holm–Sidak method or the logrank Mantel–Cox test. *p* < 0.05 was considered to indicate statistical significance.

## 3. Results

### 3.1. RABV Infection Enhanced the AAK1 Expression

**Figure 1:**
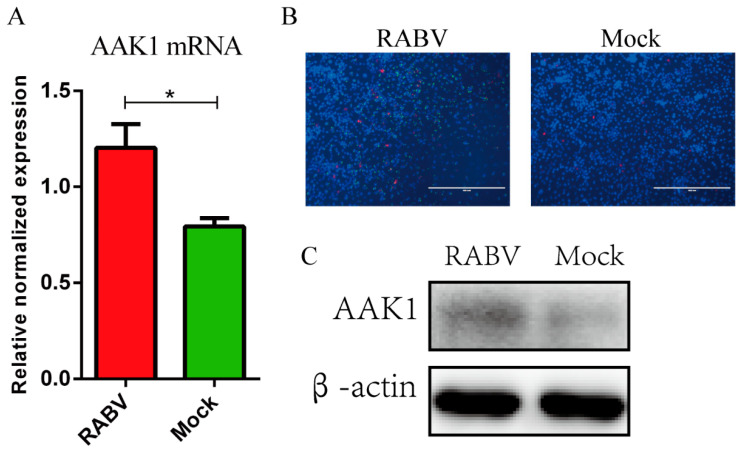
RABV proliferation increases AAK1 expression. N2a cells were infected with RABV rHEP-GFP at an MOI of 1 and incubated at 37 °C. At 24 h post infection (hpi), the inoculated N2a cells were then collected for analysis of AAK1 expression as described in the Materials and methods using different experiments. (**A**) AAK1 mRNA expression by qRT-PCR. (**B**) AAK1 expression by iFA (red for AAK1 and green for RABV N). (**C**) AAK1 protein expression by Western blotting. The AAK1 mRNA levels were normalized to GAPDH. Data represent the mean ± SD, *n* = 3. Asterisks indicate significant differences between the groups calculated using Student’s *t*-test (* *p* < 0.05).

Our previous study showed that rabies viruses (HEP-Flury strain and rHEP-dG strain) proliferation increased AAK1 expression in N2a cells by iTRAQ protein profile analysis [27]. To confirm these results, N2a cells were infected with RABV rHEP-GFP, which carries a green fluorescent protein gene between the N and P genes [29], to investigate the expression of AAK1. As shown in Figure 1A, RABV significantly increased the mRNA transcription of *AAK1* at 24 hpi. Additionally, indirect fluorescent antibody assays demonstrated upregulated expression of AAK1 in N2a cells infected with RABV (Figure 1B). Western blot analysis further confirmed the upregulated expression of AAK1 protein expression in N2a cells infected with RABV compared to the levels detected in mock infected cells (Figure 1C). Thus, RABV proliferation significantly enhanced AAK1 expression. Therefore, we speculated that AAK1 is involved in RABV infection.

### 3.2. Downregulation of AAK1 Expression Decreased RABV Infection in N2a Cells

**Figure 2:**
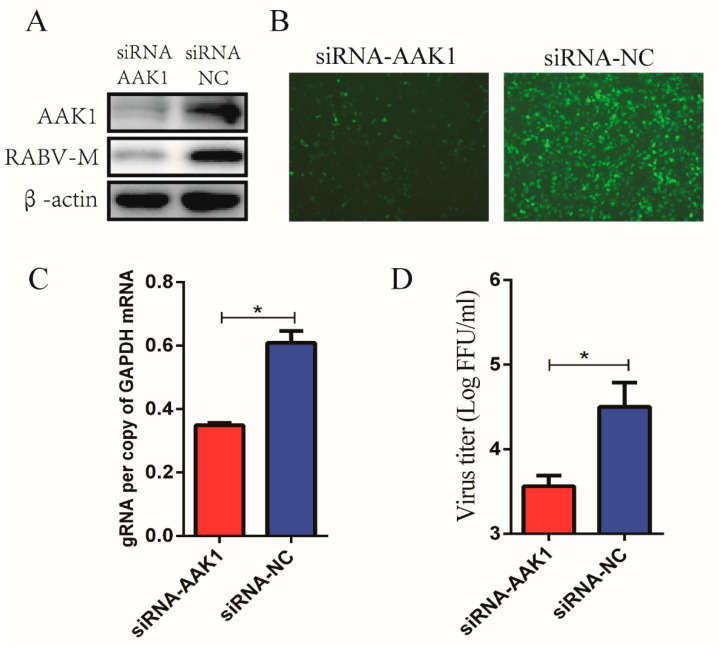
Attenuated AAK1 expression decreases RABV infection in N2a cells. N2a cells were transfected with AAK1-targeting siRNAs or negative control (NC) siRNAs and infected with RABV rHEP-GFP at an MOI of 1. At 24 hpi, AAK1 expression and RABV infection were investigated. (**A**) Expression of AAK1 and RABV M proteins in transfected N2a cells was analyzed using Western blotting with anti-AAK1 and anti-RABV M antibodies. (**B**) Analysis of GFP expression in transfected N2a cells by fluorescence microscopy. (**C**) RABV genomic RNA (gRNA) in transfected N2a cells was determined using qRT-PCR. (**D**) Virus titers in supernatants of transfected N2a cells after RABV infection by direct fluorescent antibody assays (dFA). The gRNA levels were normalized to GAPDH. Data represent the mean ± SD, *n* = 3. Asterisks indicate significant differences between the groups calculated using Student’s *t*-test (* *p* < 0.05).

To confirm the role of AAK1 in RABV infection, N2a cells were transfected with AAK1-targeting siRNAs to knock down AAK1 expression prior to infection with rHEP-GFP. Western blot analysis showed that transfection with AAK1-targeting siRNAs decreased the expression of AAK1 and RABV-M protein in N2a cells (Figure 2A). Additionally, GFP expression was decreased in N2a cells after AAK1 knockdown (Figure 2B). Furthermore, RT-qPCR demonstrated that attenuated AAK1 expression significantly decreased genomic RNA (gRNA) synthesis in N2a cells (Figure 2C). Subsequently, as expected, AAK1 knockdown decreased the rHEP-GFP virus titers in N2a cells (Figure 2D). These results indicated that AAK1 plays an important role in RABV infection.

### 3.3. AAK1 Knockdown Decreased RABV Infection in BHK-21 Cells

**Figure 3:**
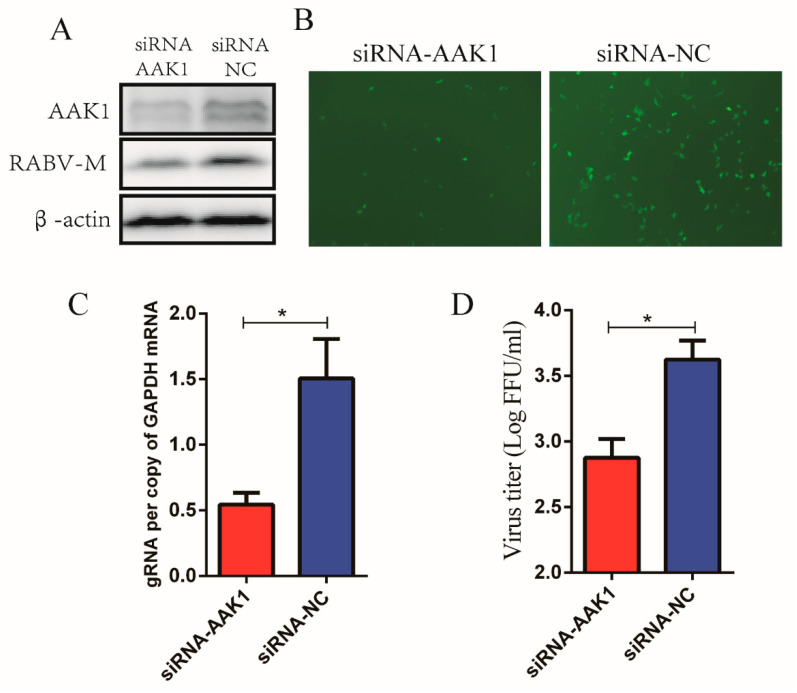
Attenuated AAK1 expression decreases RABV infection in BHK-21 cells. BHK-21 cells were transfected with AAK1-targeting siRNAs or NC siRNAs and infected with RABV rHEP-GFP to investigate AAK1 expression and RABV infection. (**A**) Expression of AAK1 and RABV M proteins in transfected BHK-21 cells was analyzed using Western blotting with anti-AAK1 and anti-RABV M antibodies. (**B**) Analysis of GFP expression in transfected BHK-21 cells by fluorescence microscopy. (**C**) RABV gRNA levels in transfected BHK-21 cells were determined using qRT-PCR. (**D**) Virus titers in supernatants of transfected BHK-21 cells after RABV infection by dFA. The gRNA levels were normalized to GAPDH. Data represent the mean ± SD, *n* = 3. Asterisks indicate significant differences between the groups calculated using Student’s *t*-test (* *p* < 0.05).

To investigate whether the effect of AAK1 on RABV infection is cell line-dependent, BHK-21 cells were transfected with AAK1-targeting siRNAs to knock down AAK1 expression prior to infection with rHEP-GFP. Western blot analysis confirmed that the expression of AAK1 and RABV-M decreased in BHK-21 cells after transfection with AAK1-targeting siRNAs (Figure 3A). Additionally, GFP expression decreased in BHK-21 cells after AAK1 knockdown (Figure 3B). qRT-PCR analysis showed that attenuated AAK1 expression still significantly decreased RABV gRNA synthesis in BHK-21 cells (Figure 3C). Furthermore, AAK1 knockdown decreased the rHEP-GFP virus titers in BHK-21 cells (Figure 3D). Therefore, these results indicated that the effects of AAK1 on RABV infection are independent from the cell lines used.

### 3.4. AAK1 Knockout Inhibits RABV Infection

**Figure 4:**
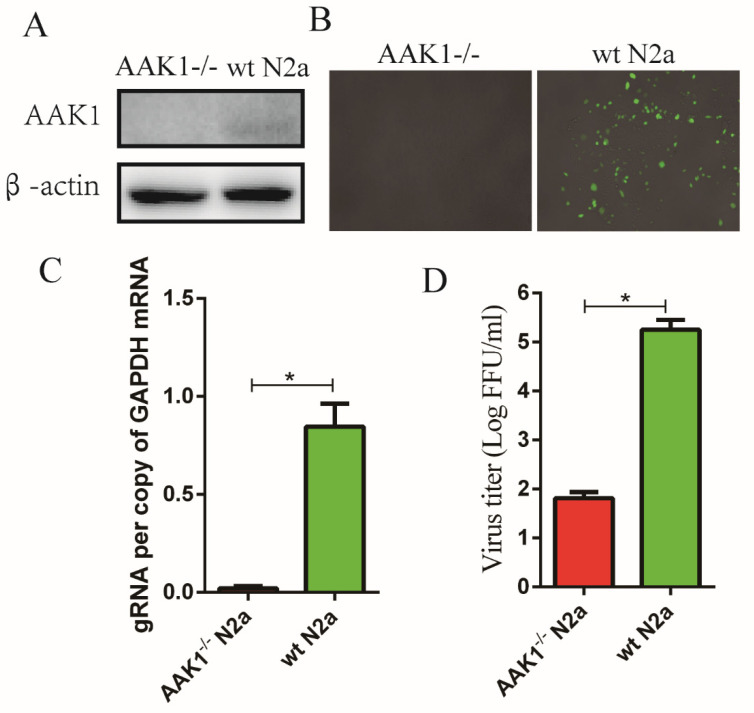
AAK1 knockout inhibits RABV infection. AAK1 was knocked out in N2a cells using CRIPR-Cas9 technology as described in the Materials and methods. (**A**) AAK1 expression was investigated by Western blotting. AAK1^-/-^ N2a and wt N2a cells were infected with RABV rHEP-GFP at an MOI of 1 for 24 h. (**B**) Analysis of GFP expression in AAK1^-/-^ N2a and wt N2a cells by fluorescence microscopy. (**C**) RABV gRNA levels in AAK1^-/-^ N2a and wt N2a cells were determined by qRT-PCR. (**D**) Virus titers in supernatants of AAK1^-/-^ NA and wt N2a cells after RABV infection (dFA). The gRNA levels were normalized to GAPDH. Data represent the mean ± SD, *n* = 3. Asterisks indicate significant differences between the groups calculated using Student’s *t*-test (* *p* < 0.05).

To further confirm that RABV infection was dependent on AAK1, AAK1 was knocked out in N2a cells using CRIPR-Cas9 technology prior to infection with rHEP-GFP. Complete knockout of AAK1 was confirmed by Western blot (Figure 4A) and no fluorescence was detected in AAK1^-/-^ N2a cells (Figure 4B). Additionally, almost no RABV gRNA were detected in infected AAK1^-/-^ N2a cells by qRT-PCR (Figure 4C). The RABV virus titer in infected AAK1^-/-^ N2a cells was significantly lower after infection than in wild-type N2a cells (Figure 4D). A small number of RABV virus were still detected in the AAK1^-/-^ N2a cell culture supernatants, possibly due to binding of residual RABV to the surface of cells. These results indicated that RABV infection is dependent on AAK1.

### 3.5. Inhibition of AAK1 Kinase Activity Decreases RABV Infection

**Figure 5:**
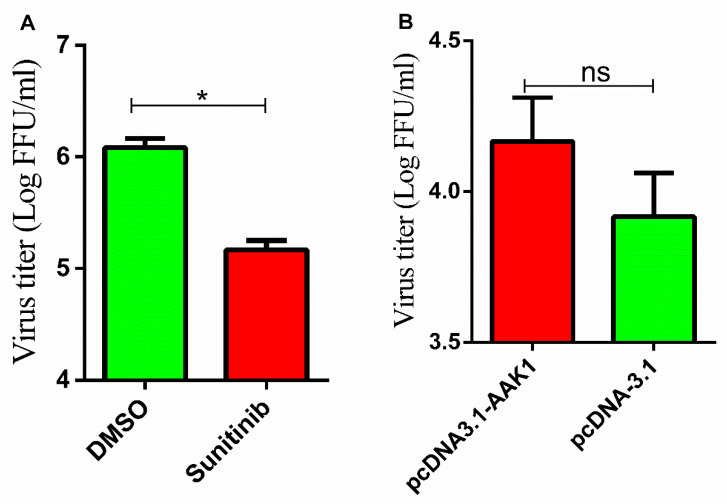
(**A**) Inhibition of AAK1 kinase activity decreases RABV infection. N2a cells were treated with 10 μM sunitinib (AAK1 inhibitor) or an equal volume of DMSO (2 μL) followed by infection with the RABV CVS-11 strain. The supernatants were harvested at 24 hpi and the virus titers were determined using dFA. (**B**) AAK1 overexpression shows no effect on RABV infection. AAK1 was overexpressed in N2a cells followed by infection with the RABV CVS-11 strain. The supernatants were harvested at 24 hpi and the virus titers were determined using dFA. Data represent the mean ± SD, *n* = 3. Asterisks indicate significant differences between the groups calculated using Student’s *t*-test (* *p* < 0.05).

Previous studies showed that the kinase activity of AAK1 can be inhibited by sunitinib [24,36]. In this study, to investigate the ability of inhibition of AAK1 kinase activity to affect RABV infection, N2a cells were treated with 10 μM sunitinib [37] prior to RABV infection. As shown in Figure 5A, sunitinib treatment significantly decreased virus titers after RABV infection, indicating that AAK1 kinase activity plays an important role in RABV infection.

### 3.6. AAK1 Overexpression Does Not Affect RABV Infection

To further investigate the role of AAK1 in RABV infection, AAK1 was overexpressed in N2a cells by transfection with p3.1-AAK1 prior to RABV infection. As shown in Figure 5B, the virus titers were not significantly increased after AAK1 overexpression (*p* > 0.05).

### 3.7. Reduced AAK1 Expression Decreases VSV Infection in N2a Cells

**Figure 6:**
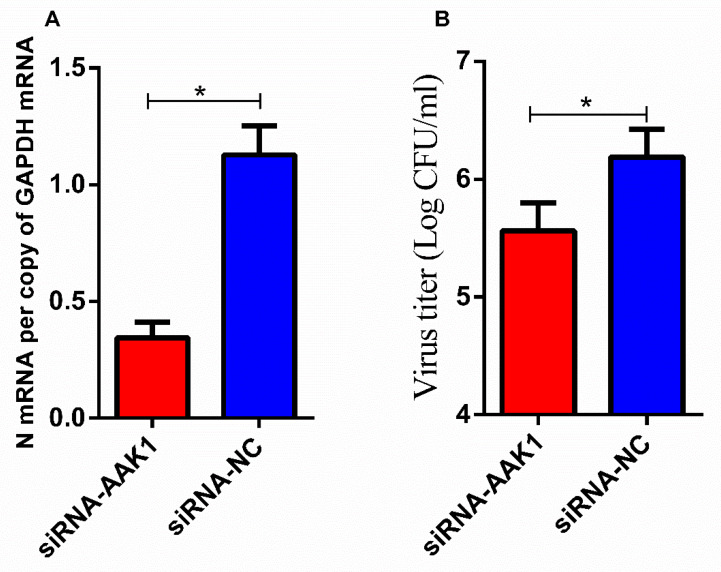
Attenuated AAK1 expression decreases VSV infection in N2a cells. AAK1 was knocked down in N2a cells by transfection with AAK1-targeting siRNAs or NC siRNAs followed by infection with VSV at an MOI of 5 for 24 h. (**A**) VSV N mRNA levels in transfected N2a cells were determined using qRT-PCR. (**B**) VSV titers in the supernatants of transfected N2a cells were determined in BHK-21 cells as described in Materials and methods. Data represent the mean ± SD, *n* = 3. Asterisks indicate significant differences between the groups calculated using Student’s *t*-test (* *p* < 0.05).

VSV is another member of the *Rhabdoviridae* family. To investigate whether AAK1 affects VSV infection, N2a cells were transfected with AAK1-targeting siRNAs to knock down the expression of AAK1 prior to VSV infection. qRT-PCR analysis showed that attenuated AAK1 expression significantly decreased VSV N mRNA levels in N2a cells (Figure 6A). As shown in Figure 6B, attenuated AAK1 expression significantly decreased virus titers after VSV infection.

### 3.8. AAK1 Inhibitor (Sunitinib) Does Not Significantly Improve the Survival Rate of Mice Following Lethal Challenge with RABV

**Figure 7:**
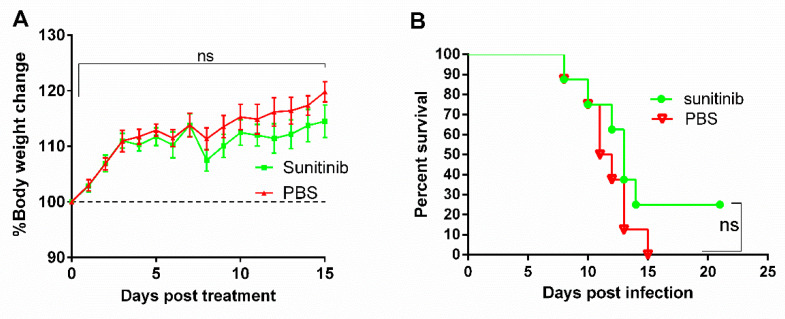
(**A**) Effects of sunitinib on mouse body weight. Sunitinib was administered to KM mice by intramuscular (i.m.) injection and body weight was monitored daily for 15 days. Changes of body weight are shown as the percentages compared to the body weight at day 0 (100%). Data are presented as mean values ± SD, and calculated by one-way ANOVA with the Holm–Sidak method. *n* = 6. (**B**) Challenge protection after sunitinib treatment. KM mice were challenged with CVS-11 by i.m. injection and at 2 days post infection, mice were treated with sunitinib via the i.m. route. Survival rates were recorded daily for 3 weeks and data were analyzed by the logrank Mantel–Cox test, *n* = 8. ns—non-significant differences.

Previous studies indicated that sunitinib was used in human at a concentration of 50 mg day^−1^ [38,39], while in mice at a concentration of 40 mg kg^−1^ [40,41]. Here, we chose 0.1 mg sunitinib as the experimental dose and the toxicity of sunitinib was investigated in mice. As shown in Figure 7A, 0.1 mg sunitinib did not result in loss of body weight in mice, compared to PBS treatment. Therefore, treatment of 0.1 mg sunitinib was safe for mice. To investigate whether the AAK1 inhibitor sunitinib affects RABV infection in vivo, KM mice were infected with the lethal CVS-11 strain followed by treatment with sunitinib 2 days post infection. As shown in Figure 7B, sunitinib treatment increased the survival rate (25%) of the challenged mice compared to that of the control group, although this difference did not reach the level of statistical significance (*p* > 0.05).

## 4. Discussion

At present, rabies infection cannot be treated after the manifestation of clinical signs. A comprehensive understanding of the mechanism underlying RABV infection is required to promote the development of novel treatment strategies. To further clarify the mechanisms underlying RABV infection, we previously performed a proteomics study based on iTRAQ and identified and quantified several distinct proteins [27]. We found that proliferation with both the RABV HEP-Flury and rHEP-dG strains increased AAK1 expression in N2a cells [27]. In this study, we further confirmed this result by determination of AAK1 mRNA and protein expression in N2a cells. Therefore, we speculate that AAK1 plays an important role in RABV infection. We showed that AAK1 knockdown significantly decreased RABV rHEP-GFP strain production in both NA and BHK-21 cells. Furthermore, AAK1 knockout prevented RABV rHEP-GFP strain infection. Thus, we showed that RABV infection is AAK1-dependent and occurs in a manner that is cell line-independent. In addition, sunitinib treatment of cells inhibited RABV CVS-11 strain infection, which further indicates that AAK1 contributes to RABV infection in a RABV strain-independent manner. However, AAK1 overexpression did not significantly increase RABV infection. We speculate that AAK1 is essential to RABV infection via a mechanism that is not dose-dependent. In addition, the study indicated that AAK1 could be localized to MHC I through an interaction with autophagy associated proteins [42]. Our study and other studies found that RABV proliferation induced autophagy in host cells [43,44]. Thus, RABV-induced autophagy may regulate AAK1 expression. The mechanism by which RABV infection inversely increased AAK1 expression requires further investigation.

RABV attachment to host cells depends on several receptors [4,5,6,7]. Subsequently, RABV is internalized by the host cells by clathrin-mediated endocytosis [8,10]. Studies have confirmed that clathrin-mediated endocytosis of RABV is actin-dependent [11,45]. AAK1 regulates clathrin-mediated endocytosis by phosphorylating the mu 2 subunit of the AP2 complex [22]. In addition, AAK1 regulates Numb function at an early step in clathrin-mediated endocytosis [23]. Therefore, we speculate that AAK1 affects RABV infection through the phosphorylation of AP2 and Numb. Previous studies have confirmed that RABV infection is dependent on the phosphorylation of AP2 by AAK1 [26]. Therefore, the loss of AAK1 might lead to significant lower number of infected cells or viral particles within an infected cell, which results in decreased gRNA and M protein synthesis and subsequently, decreased viral titers.

VSV is also a rhabdovirus and internalization occurs through clathrin-mediated endocytosis in an actin-dependent manner [46]. In this study, we found that decreased AAK1 expression attenuated VSV titers and transcription, indicating that AAK1 also plays an important role in VSV infection. Therefore, our findings indicate that rhabdovirus infection is AAK1-dependent and implicate AAK as a novel target to inhibit viral infection. In this study, mice were treated with sunitinib at 2 days post infection rather than pre-exposure. We found that the AAK1 inhibitor sunitinib did not significantly improve the survival rate of mice through the i.m. injection following a lethal challenge with RABV (*p* > 0.05). However, further work will be performed based on the formulation of sunitinib, solvent, administration route, and doses of sunitinib or other AAK1 inhibitors to further improve the therapy effect of post exposure of rabies.

In summary, we further confirmed that RABV infection depends on AAK1. In addition, AAK1 also plays an important role in VSV infection.
